# THz characterization and demonstration of visible-transparent/terahertz-functional electromagnetic structures in ultra-conductive La-doped BaSnO_3_ Films

**DOI:** 10.1038/s41598-018-22038-w

**Published:** 2018-02-23

**Authors:** Sara Arezoomandan, Abhinav Prakash, Ashish Chanana, Jin Yue, Jieying Mao, Steve Blair, Ajay Nahata, Bharat Jalan, Berardi Sensale-Rodriguez

**Affiliations:** 1 Department of Electrical and Computer Engineering, The University of Utah, Salt Lake City, UT 84112 USA; 2 Department of Chemical Engineering and Materials Science, University of Minnesota – Twin Cities, Minneapolis, MN 55455 USA

## Abstract

We report on terahertz characterization of La-doped BaSnO_3_ (BSO) thin-films. BSO is a transparent complex oxide material, which has attracted substantial interest due to its large electrical conductivity and wide bandgap. The complex refractive index of these films is extracted in the 0.3 to 1.5 THz frequency range, which shows a metal-like response across this broad frequency window. The large optical conductivity found in these films at terahertz wavelengths makes this material an interesting platform for developing electromagnetic structures having a strong response at terahertz wavelengths, i.e. terahertz-functional, while being transparent at visible and near-IR wavelengths. As an example of such application, we demonstrate a visible-transparent terahertz polarizer.

## Introduction

Transparent electronics have garnered significant attention since the introduction of the first transparent thin film transistor^1–3^. In terms of commercial products, the market has been steadily flourishing, with demand being mostly led by the photovoltaic and the optoelectronic industries. Furthermore, these materials are expected to be convenient alternatives to conventional metals as well as doped-semiconductors to develop fully transparent devices. Several types of transparent conductive oxides (TCOs) have been introduced and characterized for different applications in areas including plasmonics and photovoltaics. These include tin-doped indium oxide (ITO), gallium-doped zinc oxide (GZO), and aluminum-doped zinc oxide (AZO), among others^4^. Also, because of their large transmission in the near-infrared (IR) and visible spectral ranges, TCOs can be employed as contacts in terahertz (THz) optoelectronic devices, THz photoconductive antennas, photo-active metamaterial structures, plasmon-assisted spectroscopy of materials, etc.^5–7^; applications where the underlying material needs to be accessed optically. By virtue of being transparent at visible frequencies, these materials can be used to design structures whose optical response is encrypted at THz frequencies. On the other hand, spectroscopic characterization of these materials at terahertz frequencies reveals its high-frequency AC electronic transport properties, which enables greater understanding of carrier dynamics in these materials and thus, creates opportunities for developing devices that broaden the range of possible applications^8–10^.

With the rapid proliferation of optoelectronic devices and a need for improved performance, demand for highly conductive and cost-effective TCOs has been on the rise. Among the TCOs demonstrated thus far, ITO has been shown to possess the largest conductivity of ~10^4^ S/cm, where indium oxide is degenerately alloyed with tin (*n* ~ 10^20^ cm^−3^)^11,12^. However, future use of ITO may face a bottleneck due to the potential scarcity of indium. In this regard, doped zinc-oxide compounds have been proposed as potential replacements for ITO; however, these materials suffer from a much lower electrical conductivity^13^. Recently, thin films of ultra-conductive BaSnO_3_ (BSO) doped with lanthanum have been demonstrated and characterized^14–18^ with mobility reaching as high as 183 cm^2^/Vs^14^. Among various thin film approaches, films grown by hybrid molecular beam epitaxy exhibit the highest room-temperature conductivity of ~10^4^ S/cm at carrier concentration of 9.2×10^20^ cm^−3^ ^19^. High mobility in BSO at room-temperature has been attributed to low electron effective mass and to weak phonon scattering. These characteristics, combined with a wide band-gap of 3 eV, an ultra-high conductivity and relatively high mobility in thin films, make BSO a strong candidate as a transparent conducting oxide. Further characterization is required to reveal the true potential of this material for different applications. THz frequencies occupy a crucial region of the electromagnetic spectrum; introduction of ultra-high conductive TCOs as a building block for visible-transparent THz devices would enrich the emerging technologies in this field.

Here, we present a comprehensive study on the THz properties of ultra-conductive La-doped BSO films grown on (La_0.3_Sr_0.7_)(Al_0.65_Ta_0.35_)O_3_ LSAT(001). Details of the film growth, structural and electronic characterizations have been previously discussed^19–21^. We analyzed three sets of samples that exhibit different conductivity levels achieved by keeping a constant dopant density and varying cation stoichiometry. The optical transparency of the films at visible wavelengths was studied using visible spectroscopy while the THz properties and high-frequency carrier transport properties were characterized using time-domain THz spectroscopy. We extracted the optical properties of this material by fitting the data to proper models. Finally, we demonstrate a THz polarizer as a proof-of-concept visible-transparent THz-functional electromagnetic structure. The extracted results are compared with those reported in the literature from THz studies in ITO. Our results reveal BSO as an efficient visible-transparent THz-functional material in addition to establishing new avenues of research in this novel wide band-gap oxide films as well as new potential TCO applications.

## Experimental

BSO/LSAT(001) samples were prepared using a hybrid molecular beam epitaxy (MBE) technique^20,21^. Epitaxial films were grown on 5 mm × 5 mm, 0.5 mm thick LSAT (001) substrates. The dopant density in these films were kept constant by keeping the La cell temperature fixed during growth of active layer. Three samples with different cation stoichiometry were analyzed, which resulted in different carrier densities. For *Sample #1*, a 49 nm thick La-doped BSO film was grown on top of 49 nm thick BSO with nominally stoichiometric composition. For *Sample #*2, La-doped BSO(46 nm)/BSO (46 nm) film was grown with intentional barium deficiency. Finally, for *Sample #3*, La-doped BSO (48 nm)/BSO (48 nm) film was grown with intentional tin deficiency. Table 1 summarizes the DC transport measurements for three samples, which were obtained from Hall measurements. The samples underwent rapid thermal annealing at 800 °C for two minutes prior to the transport measurements. As shown in Table 1, stoichiometric La-doped BSO (*Sample #1*) showed the highest electron mobility, 86 cm^2^/Vs, the highest conductivity, and the highest carrier concentration. *Samples #2 and #3* with barium and tin deficiencies showed mobilities of 71 and 17 cm^2^/V.s, respectively, consistent with higher disorder due to non-stoichiometry^21^.

**Table 1:** DC extracted parameters for the analyzed BSO samples.

	Thickness (nm)	Carrier concentration (cm^−3^)	Mobility (cm^2^/V.s)	Resistivity (Ω.cm)
Sample #1 (stoichiometric)	49	6.75 × 10^20^	86	1.06 × 10^−4^
Sample #2 (Ba deficient)	46	3.73 × 10^20^	71	2.36 × 10^−4^
Sample #3 (Sn deficient)	48	1.27 × 10^20^	17	2.93 × 10^−4^

Films with similar structural and electrical characteristics were also grown on STO (001) substrates. For THz measurements LSAT was employed because STO exhibits a strong phonon absorption in the THz frequency range. In addition to being transparent at visible wavelengths, LSAT shows negligible absorption over the THz frequency range investigated here^22,23^. However, since the only LSAT substrates available were single-side polished, STO-grown samples were employed for measurements at visible frequencies. Thus, samples grown on two-side polished STO substrates were characterized using visible light spectroscopy in order to determine the transparency of the processed BSO thin films. In the sample under study, the thickness of the stoichiometric La-doped BSO film is 132 nm and the conductivity extracted from DC transport measurement was found to be 6.85 × 10^5^ S/m, which was comparable to the conductivity values observed in samples grown on LSAT substrates.

**Figure 1:**
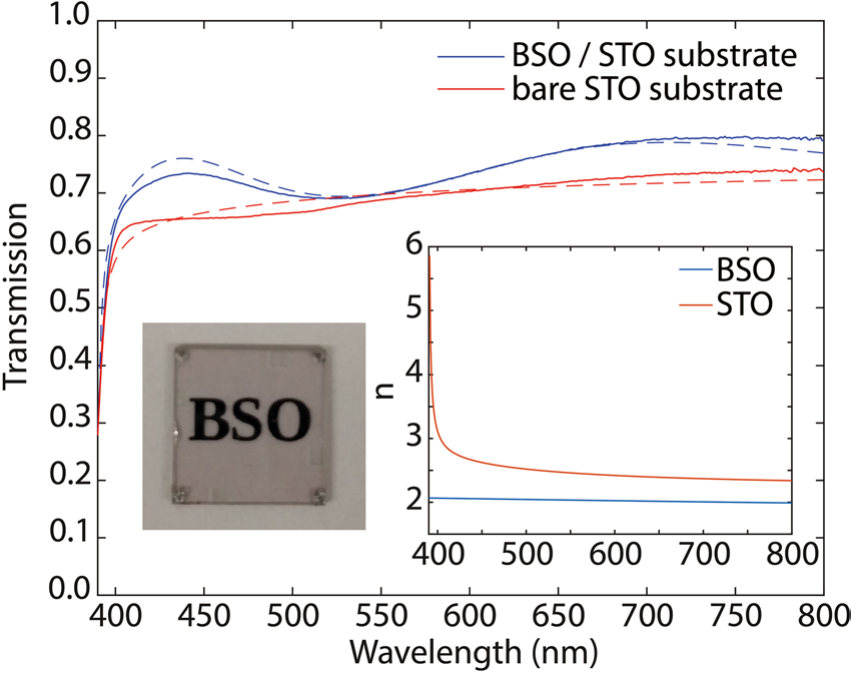
Measured optical transmission through a 132-nm film of stoichiometric La-doped BSO with a 132 nm buffer BSO layer grown on a STO substrate (blue trace) as well as optical transmission through a STO bare substrate (red trace) in the visible spectrum. Fitted curves for the measured data to the model described in the *Methods section* are indicated by dashed lines. The right inset shows the extracted refractive indices for BSO and the STO substrate extracted from this fitting. The left inset shows a picture of one of the analyzed samples showing its transparency.

Figure 1 shows the transmission spectrum for (*i*) a La-doped BSO-film grown on STO, and (*ii*) a bare STO substrate. Measurements were normalized to transmission through air. *The results indicate that the transmission for the BSO sample on STO is larger than that through the STO substrate. This can be understood as the BSO film acting as an antireflection coating*. The insets in Fig. 1 depict an optical image of the analyzed La-doped BSO sample and the extracted refractive indices for BSO and the STO substrate by fitting of the transmission data in the visible spectrum to a proper model^24^. Also, depicted in Fig. 1 is the fitting of the measured data to this model (dashed curves), showing an excellent agreement with the experiments as well as explaining the anti-reflection coating effect. The above results confirm that the La-doped BSO films are transparent across the entire visible range. The refractive index was found to be ~2 across a broad wavelength range from 400 to 800 nm, in agreement with previous reports^25,26^.

## Results and Discussion

For the purposes of THz characterization, we employed the THz time-domain spectroscopy (THz-TDS) technique as our primary characterization tool. This technique is unique, because it allows for measurement of the THz electric field (i.e. both the amplitude and phase) through the BSO/LSAT samples. By normalizing the measured spectral response to that of the substrate, we were able to independently resolve the contribution of the conductive La-doped BSO film. The normalized transmission data was used to directly extract the complex refractive index of the conductive film (as described in the *Methods* section).

**Figure 2:**
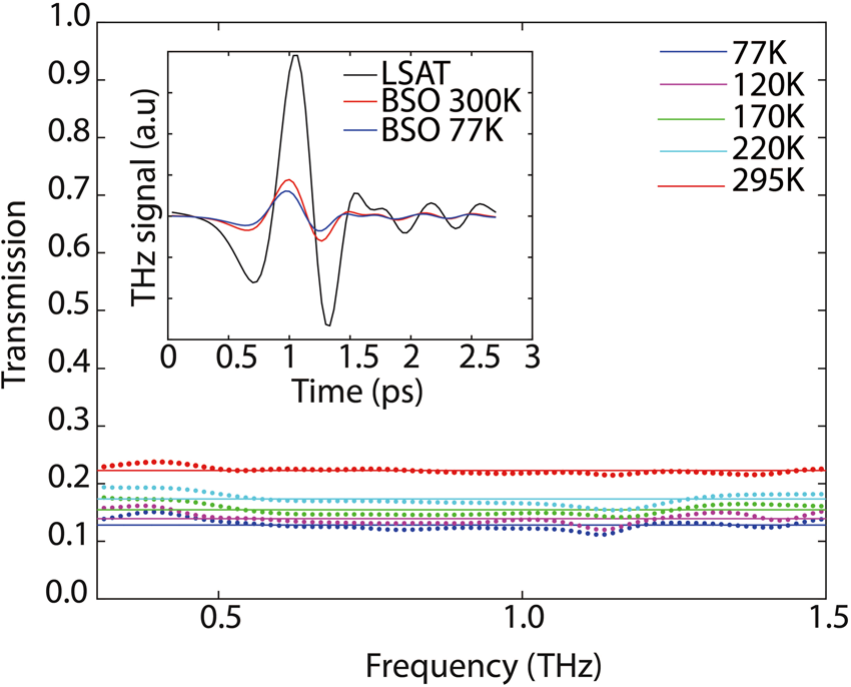
Measured terahertz transmission through Sample #1. The transmission is normalized to that of a bare substrate at each temperature. The experimental data points are indicated with dots, whereas fits to the model discussed in methods section are depicted with solid lines. The inset shows the measured terahertz pulse through the LSAT substrate at room-temperature as well as the pulses through Sample #1 at room-temperature and 77 K.

Figure 2 depicts the normalized transmission for *Sample #1* at different temperatures (77 K, 120 K, 170 K, 220 K and 295 K) in the 0.3 to 1.5 THz frequency range. The inset in Fig. 2 shows the time domain pulses measured for the reference LSAT substrate and the attenuated transmitted signal through the BSO/LSAT sample (at room temperature and 77 K). Frequency domain transmission properties are extracted from the Fourier transform of the normalized THz transmission through the substrate, allowing for extraction of the THz conductivity. As Fig. 2 shows, the transmission levels decrease as the temperature is decreased. This observation indicates an increase in the film conductivity as the temperature is decreased. Furthermore, the transmission is relatively flat in this frequency range. This observation is indicative of a short carrier momentum relaxation time; even with an increase in mobility at low temperatures, the momentum relaxation time is still relatively short and does not lead to spectral signatures in the analyzed frequency range.

**Figure 3:**
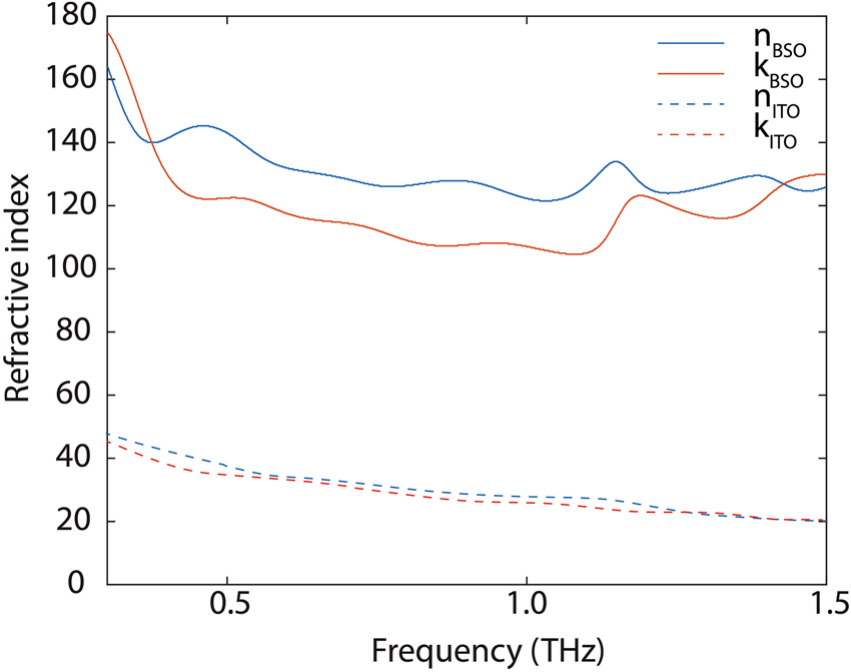
Real (*n*) and imaginary (*k*) part of refractive index vs. frequency for *Sample #1*. These parameters are directly extracted from the complex TDS transmission. In addition to the extracted values for our La-doped BSO sample (49 nm thick film), data reported in the literature^9^ for an ITO film (345 nm thick) is also depicted in the plot. In both cases, a metal-like response is observed. The conductivity levels observed in the analyzed BSO samples are on par with the best results reported in the literature for ITO^29^, and larger than those for the samples studied by terahertz spectroscopy in ref.^9^.

**Figure 4:**
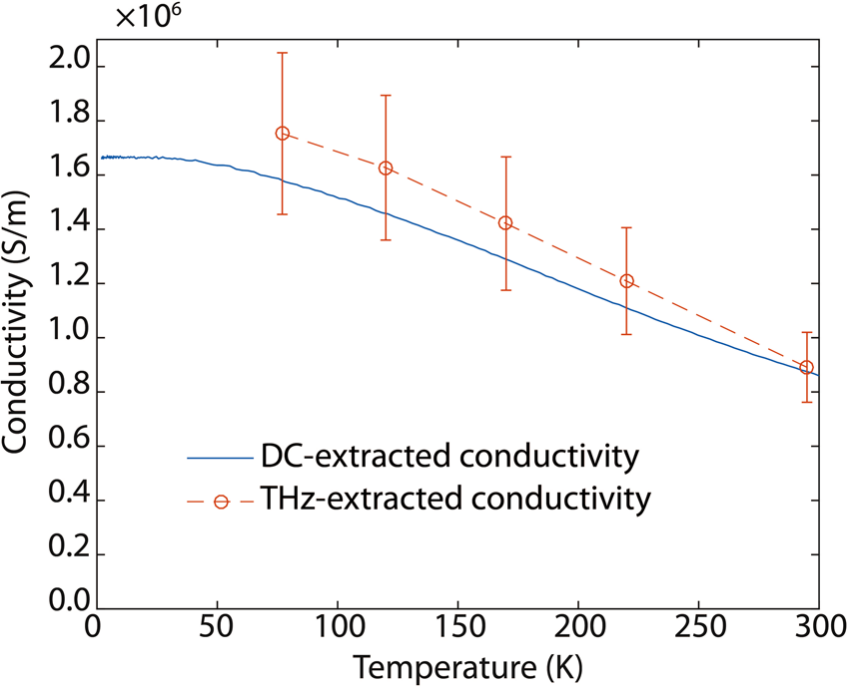
THz-extracted and DC-extracted conductivity vs. temperature for *Sample #1*. THz-extracted conductivity levels from TDS measurements statistically agree with those observed in DC measurements; this observation is different to our previous observations in complex oxide 2DEGs, where a much larger THz-extracted conductivity was observed.

From the transmission data, we extracted the refractive index and absorption coefficient following the procedure discussed in the *Methods* section. Figure 3 shows these extracted values, which were found to be relatively high when compared to reported values for ITO^9^. The behavior of these parameters, together with the flat spectral response obtained in the THz-TDS measurements, see Fig. 2, is an indication of *metal-like behavior* in these degenerate-doped semiconductors. We can also extract the THz-extracted AC conductivity for the film from the transmission data^27^. Figure 4 depicts the AC conductivity at THz frequencies for *Sample #1*, as a function of temperature. Each transmission measurement was normalized to the measured transmission through the LSAT substrate at corresponding temperatures. The increase in conductivity with the decrease in temperature can be attributed to the decrease in phonon scattering^19^. The THz-extracted AC conductivity values at each temperature shown in Fig. 4 are statistically consistent with those observed in DC measurements. This differs with our previous observations in complex oxide 2DEGs^28^, where much larger conductivity levels were extracted in the THz-measurements than in DC. In contrast to our previous work^28^, the largest differences between THz-extracted and DC-extracted conductivities were observed in non-stoichiometric samples, across all the analyzed samples in this work (*Samples #1*, #2, & *#3*), no-statistical differences were observed between DC-extracted and THz-extracted conductivity levels.

In our previous work^28^, we reported on THz characterization of the two-dimensional electron gas (2DEG) formed at the interface between polar/non-polar complex oxides (e.g. NTO/STO). By contrasting results obtained from THz and DC characterization techniques, we showed that the electrical properties of the 2DEG were significantly affected by defects and scattering at the interface. Our measurements demonstrated 3–6 X enhancement in the THz-extracted, zero-frequency AC conductivities in these samples, with respect to their DC conductivities^28^. Such an enhancement was attributed to the fact that the characteristic length over which transport was probed in terahertz spectroscopy was much smaller than the mean free path between defect-induced scattering events^28^. Based on the extracted mobility levels, we estimated this characteristic length to be of the order of 10’s of nm^28^. However, in the BSO thin-films analyzed in the current work, the mobilities extracted from Hall-effect measurements are larger, by more than one order of magnitude, than those observed in 2DEGs in NTO/STO. From this perspective, we expect the THz-probe length to be on the order of 100’s of nm in the BSO samples studied here. Thus, as a result of (*i*) a longer characteristic length at which terahertz transport is probed in these samples having larger mobility, and (*ii*) a longer mean free-path between scattering events that leads to a larger mobility in these samples, no substantial differences between DC-extracted and THz-extracted conductivity levels are expected in the BSO films. Another important factor to be considered here is that free carriers in the NTO/STO heterostructure were confined to a quasi-two-dimensional interface; however, carriers here are present in a three-dimensional BSO layer. Finally, (*iii*) the additional degree of freedom for carrier transport as a result of this increased dimensionality would limit the impact of scattering by structural defects in these samples. Therefore, the close agreement between DC-extracted and THz-extracted conductivities across all samples can be understood on basis of a larger mean free-path between scattering events as well as on a higher carrier confinement dimensionality.

**Figure 5:**
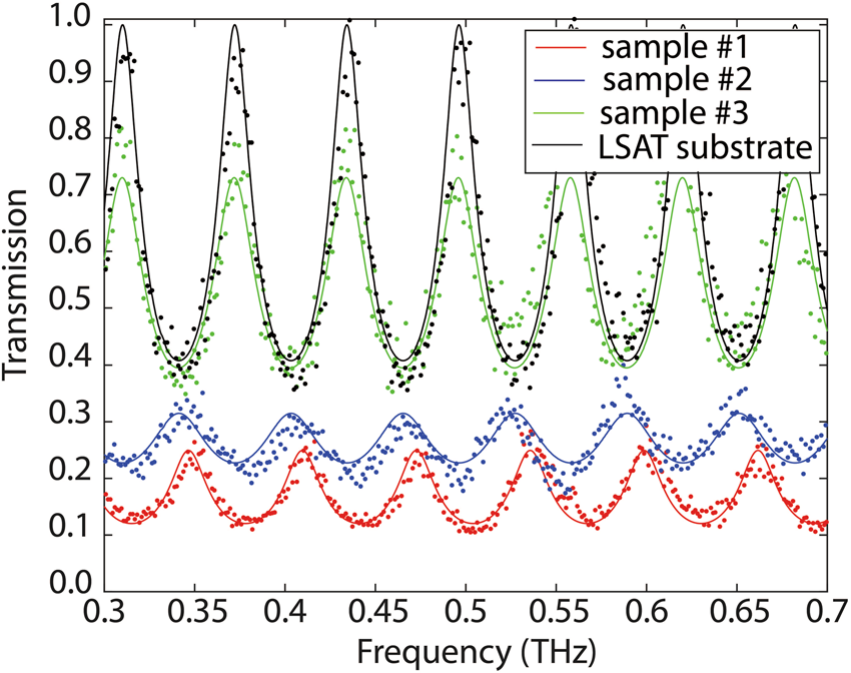
Measured CW terahertz transmission through Samples #1, #2, #3, and a bare LSAT substrate. The solid lines, represent the fitting of the measured data to the model described in *Methods* section.

In order to further validate our results and observations, we performed continuous-wave (CW) terahertz spectroscopy. In the CW-THz setup, data was obtained in the 0.3 to 0.7 THz frequency range. In Fig. 5, we show the normalized transmission spectra for *Samples* #1, #2, #3, as well as for a bare LSAT substrate. In order to properly account for the Fabry-Perot resonances resulting from the substrate, we modeled the conductive La-doped BSO film using a constant conductivity with an appropriate thickness and refractive index for the substrate. By fitting the measured data to this model, see *Methods* section, THz-extracted AC conductivities of 7.9 ± 0.2, 4.0 ± 0.6, and 0.4 ± 0.05 × 10^5^ S/m were obtained for *Samples* #1, #2, and #3, respectively. Overall, we observed close agreement between the extracted conductivity levels from THz-TDS, CW-THz, and DC measurements. It is to be noted here that, being a doped, wide band-gap semiconductor, our films demonstrate one of the highest reported conductivity levels from a TCO to-date; which is on-par of the best reported in ITO^29^. Furthermore, contrary to other highly conductive “effectively transparent” semiconductors, such as graphene, in which the high-conductivity rises from a very large electron mobility, the high conductivity in BSO is the result of an ultra-large charge density, in spite of a moderate mobility. As a result, highly conductive TCOs such as BSO have a metallic-like optical response across a wide range of terahertz wavelengths. In contrast, in high-electron mobility “transparent” semiconductors, such as high-quality graphene, the terahertz optical conductivity drops with frequency as a result of a long momentum relaxation time. From this perspective, terahertz-active and visible-transparent electromagnetic structures in BSO can provide for a broader terahertz frequency window of operation.

## Applications

**Figure 6:**
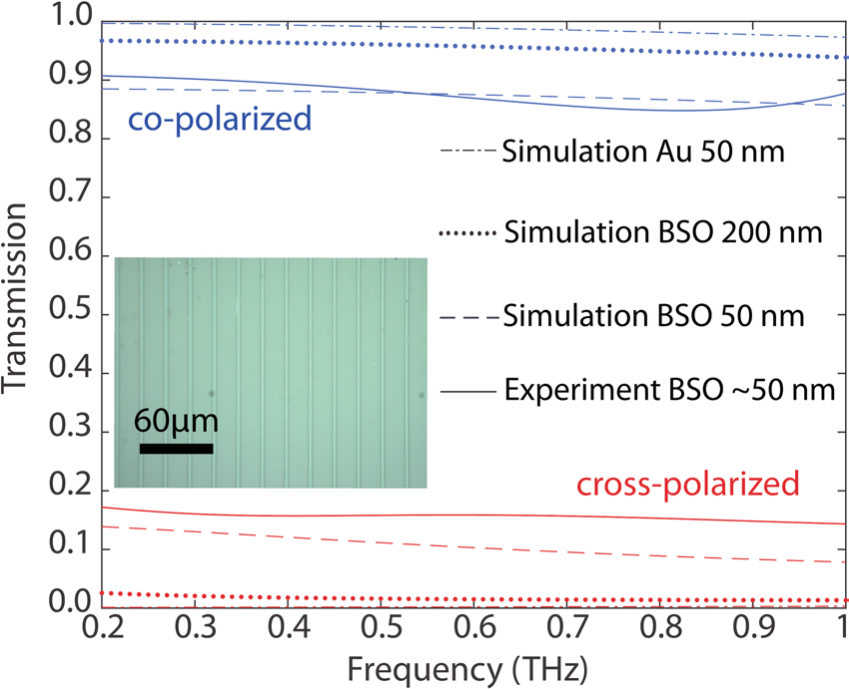
Co-polarized and cross-polarized transmission through polarizer structures. The continuous traces indicate our experimental results. Dotted and dashed curves represent modeled data. The inset shows an optical image of the fabricated sample, which is transparent to visible wavelengths.

The high carrier concentration of the La-doped BSO films leads to a metal-like response from these degenerate-semiconductors at THz frequencies, suggesting that these TCOs can be used to design terahertz-functional electromagnetic structures that are transparent to near-IR and visible wavelengths. To demonstrate this, we designed a simple grid-polarizer by patterning of the La-doped BSO films. In the case of a perfect polarizer, the co-polarized transmission should be 100%, while the cross-polarized transmission should be zero. The design geometry for the polarizer was determined with the help of full wave simulations using ANSYS HFSS. We chose a design having a wire width of 17.2 µm and 20 µm periodicity. For this purpose, *Sample #1*, i.e. the sample with the highest conductivity, was patterned using standard lithography and etching techniques (Ar ion milling). The response of the patterned-film was measured using our THz-TDS system as a function of incident-beam polarization. Depicted in Fig. 6 are the results from these measurements. For cross-polarized excitation, the transmission was 14%, while for co-polarized excitation the transmission was 88%. These experimental results were largely consistent with our simulated response, also shown in Fig. 6. In order to give further insight into the scaling of the polarizer performance with BSO-film thickness, we performed simulations for polarizers assuming 200 nm thick films by using the same THz-conductivity levels as experimentally determined in *Sample #1*. In order to provide a qualitative comparison, we also performed simulations for these polarizers using 50 nm thick gold as the conductive material. While the gold-based polarizer exhibits better performance, in terms of co- and cross-polarized transmission levels, the BSO structures are transparent at near-IR and visible wavelengths, which may be attractive for some THz applications. We found that by scaling the film thickness, a better polarizer response can be obtained.

## Conclusions

In conclusion, we have reported on terahertz characterization of La-doped BSO thin-films. Our doped BSO films show one of the largest electrical conductivities in any TCO demonstrated to date, which is on par with the best reports in ITO. Our THz measurements evidence a metallic response of these films in a broad frequency range from 0.3 to 1.5 THz. Furthermore, visible spectroscopy shows that BSO is transparent in the 400 to 800 nm wavelength window. As a result of its large broadband THz conductivity, and large visible transmission, BSO constitutes an interesting material for the design of electromagnetic structures, such as polarizers, that are functional at THz frequencies but transparent in the near-IR to visible range. From this perspective BSO is a strong candidate for future transparent devices for THz applications.

## Methods

### Film synthesis

Thin films of doped BaSnO_3_ were grown on LSAT (001) substrates using a hybrid molecular beam epitaxy approach. The method utilizes a metal-organic precursor (hexamethylditin) for tin^22^, a solid source for barium provided through an effusion cell, and an RF plasma source for oxygen. Oxygen plasma was operated at 250 W at a pressure of 5×10^−6^ Torr. Barium beam equivalent pressure (BEP) was kept constant at 5×10^−8^ Torr. Lanthanum (La) was used as an n-type dopant to make the films conducting. The dopant density was kept nominally fixed by keeping the La effusion cell temperature at 1230 °C. A substrate temperature of 900 °C was used for growing 45–50 nm thick of La-doped BaSnO_3_ layer on top 45–50 nm of undoped BaSnO_3_ buffer. Films were grown with three different tin precursor fluxes: 1.0×10^−6^ Torr (Sn-deficient), 1.4×10^−6^ Torr (stoichiometric) and 1.5×10^−6^ Torr (Ba-deficient). Structural characterization was done using *in situ* Reflection High Energy Electron Diffraction (RHEED) and X-ray Diffraction. DC transport measurements were performed in a conventional van der Pauw configuration using a Physical Property Measurement System (Dynacool). Indium was used as ohmic contacts.

### Ion Milling

BSO films were dry etched using Argon plasma in an Intlvac Ion Mill. Argon plasma was operated at 75 W with a gas flowrate of 25 sccm. Substrate was tilted at 75° with respect to the normal and cooled down to 6 °C to avoid overheating during the process. Etching was performed down to the substrate to form a polarizer structure as shown in the inset of Fig. 6.

### Terahertz spectroscopy

#### THz-TDS

the terahertz signal was generated by optical rectification in a ZnTe crystal pumped by an 810 nm amplified Ti-Sapphire laser with pulse width of 50 fs and repetition rate of 1 kHz. The terahertz signal was then focused on the samples, which were placed inside a cryostat, using two parabolic mirrors. The transmitted THz electric field was focused and sampled using an optical probe beam in a second ZnTe crystal via electro-optic sampling. The time domain terahertz waveforms were Fourier transformed to extract the frequency response of the sample.

#### THz-CW

the samples were also tested in a diode-laser-driven photomixing spectrometer (Toptica Photonics). Two lasers were detuned about a center wavelength of 1500 nm, allowing for are photomixed generation and detection of narrowband THz radiation.

### Visible spectroscopy

Visible spectroscopy was performed using a Perkin-Elmer LAMBDA 950 UV-Vis-NIR Spectrophotometer with a 150 mm PbS integrating sphere in the wavelength range from 390 to 800 nm using 1 nm steps.

### Modeling

- THz-TDS: direct extraction of the complex refractive index. The transmission through a thin optical film (medium #2) sandwiched between two thick optical materials (mediums #1 and #3) normalized to the transmission from medium #1 to #3, can be modeled by^9,30^:
  1 $${T}_{sample}(\omega )=\frac{{E}_{sample}(\omega )}{{E}_{substrate}(\omega )}=\frac{{t}_{12}{t}_{23}{e}^{\frac{i({n}_{2}-1)d\omega }{c}}}{{t}_{13}}FP(\omega ),$$
  here *n*_*2*_ is the refractive index of medium #2, *d* is its thickness, *c* is the speed of light, *ω* is angular frequency, t_ij_ is transmission from medium i to medium j and is defined as ${t}_{ij}=\frac{2{n}_{i}}{{n}_{i}+{n}_{j}}$, and FP(ω) is a Fabry-Perot term set by the multiple reflections inside the optically thin slab. This last term is defined as:
  2 $$FP(\omega )=\frac{1}{1-{r}_{21}{r}_{23}{e}^{j2{n}_{2}d\omega /c}},$$
  where r_ij_ is the reflection from the interface of medium i and j and defined as ${r}_{ij}=\frac{{n}_{i}-{n}_{j}}{{n}_{i}+{n}_{j}}$. Here, medium #1 is air and medium #3 is the LSAT substrate. By fitting the transmission data to this model, the refractive index of the BSO layer can be extracted at each frequency.
- THz-TDS: extraction of conductivity from transmission spectra. The optical conductivity of the BSO film was extracted from the transmission data by fitting to^27^:
  3 $$1-{|\frac{T}{{T}_{0}}|}^{2}=1-\frac{1}{{|1+\frac{\sigma (\omega ){Z}_{0}}{{n}_{s}+1}|}^{2}},$$
  where, Z_0_ is the characteristic impedance of free space, n_s_ is the refractive index of the substrate, and σ(ω) is an effective 2D conductivity for the La-doped BSO layer, i.e. conductivity [S/m] multiplied by thickness of the film. The above formula is valid for situations where the effective optical thickness of the film is much smaller than the relevant terahertz wavelengths, which is the case in our study. Since the measured transmittance did not exhibit significant frequency variations, the measured data was fitted to the above formula assuming a constant conductivity.
- CW-THz spectroscopy: extraction of conductivity from transmission spectra. In CW measurements, multiple reflections from the substrate need to be considered, therefore a different theoretical framework needs to be employed so to model the data. For this purpose, the ABCD matrix formalism is employed. By fitting of the experimental data to this analytical model the conductivity of the film is extracted. It is worth mentioning that since the BSO film is very conductive, we are not capable to determine its real part of permittivity employing this approach. The LSAT substrate is modelled by^31^:

4 $$\begin{array}{rcl}{(\begin{array}{ll}A & B\\ C & D\end{array})}_{LSAT} & = & (\begin{array}{ll}\cos ({{\rm{\Phi }}}_{LSAT}) & {\rm{j}}{Z}_{LSAT}\,\sin ({{\rm{\Phi }}}_{LSAT})\\ {\rm{j}}\frac{1}{{Z}_{LSAT}}\,\sin ({{\rm{\Phi }}}_{LSAT}) & \cos ({{\rm{\Phi }}}_{LSAT})\end{array})\end{array},$$

where: ${{\rm{\Phi }}}_{LSAT}=\frac{{n}_{LSAT}\omega {d}_{LSAT}}{c}$, *n*_*LSAT*_ is the refractive index of the LSAT substrate, *ω* in angular frequency, *d*_*LSAT*_ is the thickness of the LSAT substrate and *c* is the speed of light. Furthermore, ${Z}_{LSAT}=\frac{{Z}_{0}}{{n}_{LSAT}}$, where Z_0_ is the vacuum impedance. The transmission through the LSAT substrate can be extracted using the following formula^31^:

5 $$T=\frac{2}{A+\frac{B}{{Z}_{0}}+C{Z}_{0}+D}.$$

The doped BSO film can also be modelled by an ABCD matrix:

6 $$\begin{array}{rcl}{(\begin{array}{ll}A & B\\ C & D\end{array})}_{BSO} & = & (\begin{array}{ll}\cos ({{\rm{\Phi }}}_{BSO}) & {\rm{j}}{Z}_{BSO}\,\sin ({{\rm{\Phi }}}_{BSO})\\ {\rm{j}}\frac{1}{{Z}_{BSO}}\,\sin ({{\rm{\Phi }}}_{BSO}) & \cos ({{\rm{\Phi }}}_{BSO})\end{array})\end{array},$$

where: ${{\rm{\Phi }}}_{BSO}=\frac{{n}_{BSO}\omega {d}_{BSO}}{c}$, *n*_*BSO*_ is the complex refractive index of the BSO layer, and *d*_*BSO*_ is its thickness. The complex refractive index can be defined as:

7 $${n}_{BSO}=\sqrt{{\varepsilon }_{r}-j\frac{{\sigma }_{BSO}}{\omega {\varepsilon }_{0}}}\approx \frac{(1+j)}{\sqrt{2}}\sqrt{\frac{{\sigma }_{BSO}}{\omega {\varepsilon }_{0}}},$$

here: *ε*_0_ is the vacuum permittivity, and σ_BSO_ is the conductivity of the BSO film. In this case, the ABCD matrix representing the total structure can be found from the matrix multiplication of (ABCD)_total_ = (ABCD)_LSAT_.(ABCD)_BSO_. It is worth mentioning again that in the analyzed frequency ranges, the transmission was found to be constant over frequency, therefore the terahertz optical conductivity was modeled as a constant across this frequency range.
